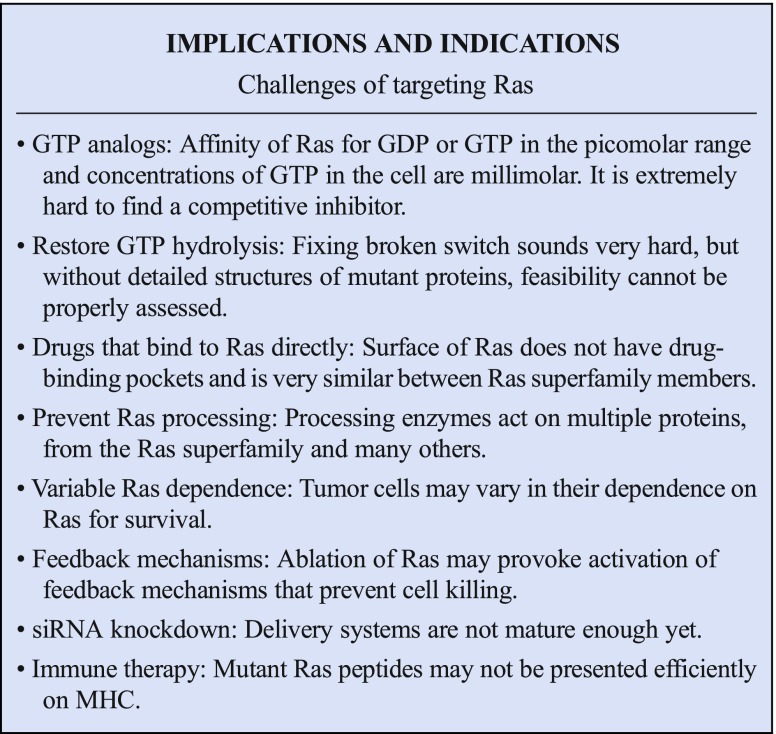# K-Ras protein as a drug target

**DOI:** 10.1007/s00109-016-1382-7

**Published:** 2016-03-09

**Authors:** Frank McCormick

**Affiliations:** UCSF Helen Diller Family Comprehensive Cancer Center, 1450 3rd Street, San Francisco, CA 94158-9001 USA

**Keywords:** K-Ras proteins, Cancer, K-Ras therapies

## Abstract

K-Ras proteins are major drivers of human cancers, playing a direct causal role in about one million cancer cases/year. In cancers driven by mutant K-Ras, the protein is locked in the active, GTP-bound state constitutively, through a defect in the off-switch mechanism. As such, the mutant protein resembles the normal K-Ras protein from a structural perspective, making therapeutic attack extremely challenging. K-Ras is a member of a large family of related proteins, which share very similar GDP/GTP-binding domains, making specific therapies more difficult. Furthermore, Ras proteins lack pockets to which small molecules can bind with high affinity, with a few interesting exceptions. However, new insights into the structure and function of K-Ras proteins reveal opportunities for intervention that were not appreciated many years ago, when efforts were launched to develop K-Ras therapies. Furthermore, K-Ras undergoes post-translational modification and interactions with cellular signaling proteins that present additional therapeutic opportunities, such as specific binding to calmodulin and regulation of non-canonical *Wnt* signaling.

## Introduction

Activating mutations of signaling molecules occur in many types of cancer, often at high frequencies [1]. Mutations in K-Ras alone account for one million deaths/year. In adenocarcinoma of the lung, the receptor tyrosine kinase (RTK) pathway, including Ras, neurofibromin, and Raf kinase, is the major oncogenic driver pathway, accounting for at least 75 % of all cases. Pancreatic cancer mutations in K-Ras account for more than 90 % of cases, while mutations in RTKs themselves are notably absent. Ras genes and other components of the Ras/RTK pathway are activated at high frequency in colorectal cancer, in various hematopoietic malignancies, and in multiple other cancers, but the contributions from different members of the Ras/RTK pathway vary dramatically, for reasons that are not understood at all. Notably, the Ras/RTK pathway is not frequently activated in breast cancers or prostate cancers, presumably because estrogen and androgen receptors provide mitogenic stimulation in these tumor types. However, the degree to which these types of cancer remain dependent on wild-type Ras proteins has not been clearly established.

Mutations in the Ras pathway also account for the relatively common syndromes neurofibromatosis type 1 (loss of function of the Ras GAP neurofibromin) and Noonan’s syndrome (activating mutations in SHP2, Sos, K-Ras, SPRED1), and rarer diseases such as Costello syndrome (germline mutations in H-Ras), cutaneous facial cardio syndrome (activating mutations in B-Raf or MEK) [2]. However, amongst the activating mutations in the pathway, K-Ras itself is the most frequent, yet it has been the least amenable to drug discovery. In this review, I will discuss the underlying reasons for this.

## The cycle of activation and inactivation

Ras proteins are binary switches: the GTP-bound form is ON, and the GDP-bound form is OFF [3]. Transition between these states occurs very slowly in the absence of any signals. Transition back from Ras-GDP to Ras-GTP occurs with similar kinetics: GDP dissociates from Ras very slowly. Once GDP has dissociated from Ras, it is replaced by rapid binding with GTP, which is typically present at a much higher concentration than GDP. These intrinsic properties are probably not relevant to Ras signaling, since both steps (GTP hydrolysis and GDP/GTP exchange) are actively regulated by other proteins. Ras proteins accumulate in their GTP states rapidly in response to signals, through the action of guanine nucleotide exchange factors (GEFs), of which the best understood is Sos [4, 5]. This multi-domain protein is recruited to activated receptors in the plasma membrane. Local proximity to Ras in the plasma membrane appears sufficient to activate Ras, based on the discovery that simply fusing a CAAX motif to the C-terminal region of Sos renders it constitutively active. Sos is certainly regulated at many other levels (phosphorylation, for example, and by allosteric effects of a second Ras binding site), but membrane translocation seems to be the fundamental driver.

Similar general principles apply to the Ras inactivation process, which is mediated by GTP hydrolysis. The intrinsic rate is very slow, with a *t*_1/2_ of hours, but this rate is increased up to 100,000× by GTPase-activating proteins (GAPs). One of the better characterized is p120GAP (RASA1): this protein, like Sos, is recruited to activated receptors and turns Ras off again through proximity in the plasma membrane. Neurofibromin is another form of GAP that plays a major role in regulating Ras in many cell types. This protein exists as a complex with SPRED proteins that are essential for localization of neurofibromin in the plasma membrane [6]. The mechanism by which this complex is regulated during signaling is not yet known. However, of all the Ras GAPs, neurofibromin is by far the most frequently mutated in human cancer, accounting for about 8 % of lung adenocarcinomas and 25 % of glioblastomas for example (Cancer Genome Research Network). In these diseases, loss of neurofibromin most likely acts as a primary driver, pheno-copying activating mutations in Ras which generally function through rendering Ras proteins insensitive to GAP action [7].

## What Ras proteins do

**Fig. 1:**
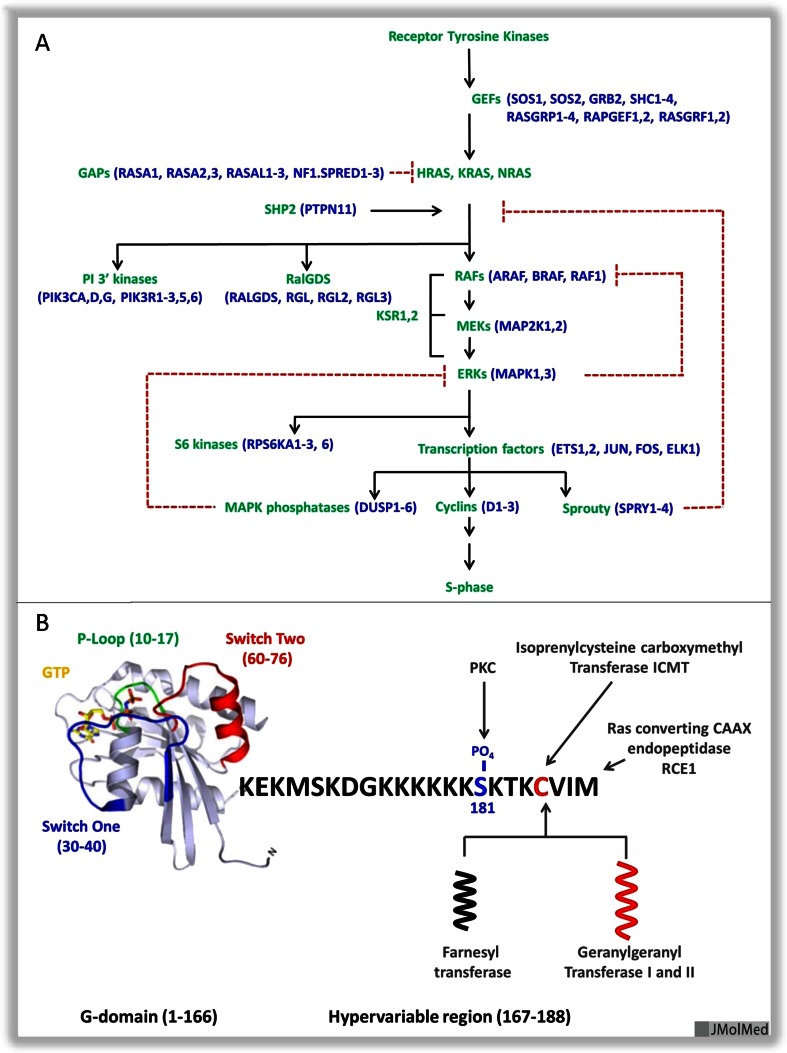
**a** The Ras MAP kinase pathway. **b** The K-Ras 4B protein

Genetic analysis of Ras function in model organisms helped enormously to delineate the Ras pathway. In *Caenorhabditis elegans* and *Drosophila melanogaster*, Ras activates the MAPK pathway (Fig. 1a). The same pathway exists in mammalian cells, though many details need to be resolved. For example, the precise roles of KSR and SHP2 are still unclear at the molecular level, and we still do not understand exactly how Ras proteins activate Raf kinase, an essential step in the Ras signaling pathway. In normal mammalian cells, and in many cancers, the Raf/MAPK pathway is indeed the major effector pathway. For example, in mouse embryonic fibroblasts (MEFs) devoid of Ras proteins, proliferation and migration can be rescued by activated alleles of Raf, MEK or ERK, but not by other candidate effectors, PI3K or RalGDS [8]. Furthermore, tumors driven by K-Ras in mouse models can be ablated by deletion of c-Raf [9]. However, it has been clear for many years that alternative effector pathways may exist: for example, in *Saccharomyces cerevisiae*, Ras proteins perform very different functions: they regulate adenylyl cyclase activity. In *Dictyostelium discoideum*, Ras proteins directly regulate PI3K and mTOR and do not control the MAPK pathway. In *Schizosaccharomyces pombe*, Ras proteins do activate a MAPK pathway, but the direct effector of Ras is a kinase that does not resemble Raf kinase at all-in overall domain structure and organization [10].

Mammalian Ras proteins in their GTP state bind and activate several other effectors in addition to Raf kinases, each of which has been shown to play a role in Ras-driven cancers. Class I PI3K bind to Ras.GTP directly [11], resulting in increased activity. Furthermore, this interaction is necessary for tumor formation and maintenance in mouse models of K-Ras lung cancers [12, 13] as well as induction of angiogenesis [14]. However, some types of cancer driven by Ras oncogenes do not appear to utilize this mechanism to activate PI3K, and more work needs to be done to determine the molecular parameters that govern this interaction. One interesting approach that supports a critical role for PI3 kinase activation in Ras carcinogenesis utilized Ras mutants that are defective in PI3K binding. These mutants are defective at tumor initiation in mouse lymphoma models: however, tumors that do emerge show elevated levels of PI3K signaling, often through loss of expression of PTEN. They seem to compensate for loss of Ras activation of PI3K by this alternative mechanism [15]. RalGDS proteins (RalGDS, RGL, RGL2, and RGL3) are also well-validated effectors of Ras signaling, though their precise role is less clear [16]. The roles of distinct Ras effectors in normal cells and in cancer have been discussed recently elsewhere [17, 18].

## The target itself

The Ras protein consists of a highly conserved G-domain and a hypervariable tail that is involved in membrane localization and, in some cases, unique functions that distinguish Ras isoforms [17]. The first 80 amino acids of the G-domain are identical between H-Ras, K-Ras, and N-Ras and contain the two regions of the Ras protein that undergo major conformational changes between the GDP-bound form and the GTP-bound form. These two regions, switch one and switch two, are the sites of binding of Ras’ effectors and GAPs. Raf kinases bind at amino acids 32--40 within switch one, PI3K, RalGDS and GAPs bind at both switches one and two (Fig. 1b). These 80 amino acids are also highly conserved between proteins of the Rap family, and proteins of the R-Ras family [19]. This is relevant to drug discovery, as these Ras relatives perform distinct functions in cell biology that appear unrelated to effector functions of the H, N, and K-Ras. For example, Rap proteins regulate multiple processes linked to actin cytoskeletal dynamics, including integrin-mediated and cadherin-mediated adhesion, all functions that are distinct from those ascribed to Ras. Rap proteins do not activate Raf kinase in vivo, even though their effector binding regions (amino acids 32–40) are identical to Ras. Indeed, Rap proteins bind Raf kinase in vitro, but fail to do so in vivo, for reasons that are not understood. Nevertheless, the possibility that drugs that bind to the Ras G-domain may also bind G-domains of Ras family members needs to be taken seriously.

The G-domain binds guanine nucleotide with picomolar affinity. These nucleotides hold the Ras protein in active or inactive states, and only cycle on and off Ras in response to signals. For this reason, the approach of blocking Ras with nucleotide competitors seems extremely challenging. Furthermore, cellular pools of GTP approach millimolar concentrations, further complicating attempts to utilize GTP or GDP competitive analogs. The high degree of conservation between members of the Ras superfamily presents another complication to this approach. While these parameters appear daunting, there may yet be opportunities to exploit nucleotide binding properties of Ras proteins therapeutically. For example, when Sos binds Ras, the off-rate for GDP increases dramatically, allowing exchange of GDP for GTP, which exists at a sufficiently high concentration to bind Ras even during this low affinity state. This might present an opportunity for competitive binding of a nucleotide analog that somehow inhibits function.

Mutations in the GTPase site prevent GAP-mediated GTP hydrolysis and reduce intrinsic GTPase activity: they also present a unique opportunity for therapeutic intervention. The G12C mutation has been targeted directly by electrophilic compounds that bind in a pocket close to G12C, or to the GTP-site itself, and covalently bind to the cysteine residue [20, 21]. These compounds lock the Ras protein in its inactive GDP state and so prevent downstream signaling. Whether similar strategies can be employed to target other mutations remains to be seen. G12D is the most common allele, but the aspartate side group offers fewer chemical options for reactive attack.

Analysis of Ras structures using NMR and molecular dynamic modeling has led to recognition that the G-domain actually exists as two lobes [22]. The first, which consists of amino acids 1–86, contains the effector binding regions switches one and two, as well as the P-loop (10–17). This lobe is identical between H-Ras, N-Ras, and K-Ras, as described above. The second lobe diverges slightly between isoforms and has been referred to as the allosteric lobe. This lobe is predicted to form direct contacts with the plasma membrane and contains sites of potential allosteric regulation by ligands such as acetate and calcium [22]. Binding of these ligands causes conformational changes which affect the conformation of switch two in the first lobe, and could therefore have an important role in signaling. These exciting discoveries suggest new possibilities for identifying small molecules that affect Ras activity.

The most direct and obvious way to attack oncogenic Ras would be to restore GAP-mediated GTP hydrolysis. This would address the primary cause of oncogenic activation. This approach seems, at first sight, to be technically out of reach, as it would require precise re-structuring of the active site, to allow the optimal orientation of the arginine finger from GAP, water molecules in the active site, the gamma phosphate of GTP and side chains of glutamine, and possibly other Ras residues. Furthermore, there may be little space in the active site for a small molecule to bind. However, no structures have been solved of oncogenic Ras mutants complexed with GAPs. Therefore, the possibility of affecting the GTPase activity of this complex should not be dismissed too quickly. Furthermore, intrinsic GTPase might be important in regulating the duration of Ras signaling, so that modest increases in this parameter could have useful therapeutic benefit [17].

## Post-translational modifications

The C-terminal CAAX motif is modified in three steps: farnesylation, proteolysis, and esterification of the C-terminal carboxylic acid (Fig. 1b). Fully processed Ras proteins derive specificity and high affinity for plasma membranes through subsequent palmitoylation (H-Ras, N-Ras, K-Ras 4A) or through association of lysine residues with membrane lipids (K-Ras4B). Membrane association is essential for Ras function: therefore, enzymes involved in Ras processing have long been evaluated as therapeutic targets [23]. Unfortunately, targeting farnesyl transferase was unsuccessful, most likely because geranylgeranyl transferase can also modify K-Ras and N-Ras proteins in the absence of farnesyl transferase activity. H-Ras proteins are not substrates for this back-up mechanism, and new efforts are underway to test farnesyl transferases in cancers driven by mutant H-Ras. The other enzymes involved in Ras processing, the Ras CAAX endopeptidase RCE1 and the isoprenylcysteine carboxy methyltransferase (ICMT) and palmitoyl transferases, have not been fully validated as suitable drug targets, because these enzymes are involved in processing multiple other proteins, or because data from knock-out experiments have produced conflicting results. However, we should not exclude the possibility that new insights into Ras processing and new chemical approaches might restore interest in targeting Ras processing, as discussed in Cox et al. [23].

A novel aspect of Ras signaling has been exploited recently as a new therapeutic strategy. This is based on new insights in intramembrane trafficking and the appreciation that plasma membranes are constantly in the process of re-cycling. Chaperone proteins are therefore required to maintain Ras proteins at the plasma membrane. PDE-delta is a farnesyl-binding protein that performs this function for K-Ras 4B and other farnesylated proteins. Inhibition of the interaction between K-Ras 4B and PDE-delta inhibits K-Ras 4B function and suggests new avenues for therapy [24].

In addition to the well-studied processing reactions at the CAAX box at the C-terminus, and at sites of palmitoylation (N-Ras, H-Ras, K-Ras 4A), Ras proteins can be modified by acetylation [25], ubiquitination [26], or, in the case of K-Ras 4B, by phosphorylation [27]. While the importance of the former modifications has been difficult to assess, the role of phosphorylation is becoming more clear. PKC promotes phosphorylation of S-181 on K-Ras 4B, resulting in an electrostatic switch that affects K-Ras 4B association with the plasma membrane and compromises K-Ras 4B’s activity [27, 28]. In addition, phosphorylation prevents association of K-Ras 4B with calmodulin [29]. As a result, CaM kinase activity is decreased, resulting in loss of signaling through the non-canonical Wnt/Ca2+ signaling pathway [30]. This promotes cancer cell stem-ness, as defined by tumor initiating potential, though the mechanism by which this is achieved is not yet clear. Agents that increase PKC activity and K-Ras 4B phosphorylation, such as bryostatin [27] or prostratin [30], interfere with K-Ras 4B signaling and prevent K-Ras-induced stem-ness and so prevent tumor initiation, in several mouse models of cancer. These data offer a novel approach to cancer therapy that is mediated by direct effects on K-Ras, albeit through pathways that appear unrelated to the traditional effector pathways shown in Fig. 1a.

## Conclusions

The challenge of targeting Ras onco-proteins effectively and safely is certainly enormous. However, the urgent clinical need is also enormous and clearly justifies renewed efforts to tackle this extremely difficult problem. The biochemical properties of the protein, including its high affinity for nucleotide and lack of druggable pockets, are part of the problem. Further difficulties, along with the subtle nature of the activating mutations, are the very high degree of similarity with other Ras proteins and Ras’ cousins. However, progress is being made through clever insights into new chemical approaches and a better understanding of biophysical properties of the protein and a new appreciation of the roles of post-translational modifications. This new momentum leads us to be cautiously optimistic. In parallel, other approaches to silence expression of Ras genes, new ways of identifying down-stream vulnerabilities, and the possibility of harnessing the immune system to attack mutant Ras proteins directly, or Ras cancers indirectly, all add to the sense that this problem will be solved and that finally patients suffering from Ras-driven cancers will benefit from revolution in cancer drug discovery and treatment that has already affected countless cancer patients whose tumors are driven by more refractory targets.